# Human lactobacilli as supplementation of clindamycin to patients with bacterial vaginosis reduce the recurrence rate; a 6-month, double-blind, randomized, placebo-controlled study

**DOI:** 10.1186/1472-6874-8-3

**Published:** 2008-01-15

**Authors:** Per-Göran Larsson, Babill Stray-Pedersen, Kjeld R Ryttig, Stig Larsen

**Affiliations:** 1 Department of Obstetrics and Gynaecology, Kärnsjukhuset, SE-541 85 Skövde, Sweden; 2 Department of Obstetrics and Gynaecology, University of Oslo, Norway Rikshospitalet/Radiumhospitalet, Oslo, Norway; 3 Farmaservice, DK-2990 Nivå, Denmark; 4 Norwegian School of Veterinary Science; also Ullevål University Hospital, Oslo, Norway

## Abstract

**Background:**

The primary objective of this study was to investigate if supplementary lactobacilli treatment could improve the initial cure rate after vaginal clindamycin therapy, and secondly, if lactobacilli as repeated adjunct treatment during 3 menstrual cycles could lengthen the time to relapse after initial cure.

**Methods:**

Women (n = 100) with bacterial vaginosis diagnosed by Amsel criteria were after informed consent offered vaginal clindamycin therapy followed by vaginal gelatine capsules containing either 10^9 ^freeze-dried lactobacilli or identical placebo capsules for 10 days during 3 menstrual cycles in a double-blind, randomized, placebo-controlled trial.

**Results:**

The initial intent to treat (ITT) analysis for the one-month cure rate was 64% in the lactobacilli group and 78% in the placebo group (p > 0.05). However, any patient with missing or unclassified smears at the initial visit who continued the study and whose next smear indicated a cure was included in the cured group; the study also excluded two of the patients in the lactobacilli group who reported that they did not take any vaginal capsules. With consideration to these population changes, the initial cure rate would be 77% in the lactobacilli group. The 76 cured women were followed for 6 menstrual cycles or until relapse within that time span. At the end of the study, 64.9% (24/37) of the lactobacilli treated women were still BV-free compared to 46.2% (18/39) of the placebo treated women. Comparison of the two groups regarding "Time from cure to relapse" was statistically significant (p = 0.027) in favour of the lactobacilli treatment. Adjuvant therapy with lactobacilli contributed significantly to avoidance of relapse with a proportional Hazard Risk ratio (HR) of 0.73 (0.54–0.98) (p < 0.05)

**Conclusion:**

The study shows that supplementary treatment combining two different strains of probiotic lactobacilli does not improve the efficacy of BV therapy during the first month of treatment, but for women initially cured, adjunct treatment of lactobacilli during 3 menstrual cycles lengthens the time to relapse significantly in that more women remained BV free at the end of the 6-month follow up.

**Trial registration number:**

ISRCTN62879834

## Background

Bacterial vaginosis (BV) is a disease with unknown aetiology, characterized by loss or reduction of lactobacilli and increased overgrowth of other bacteria. It is one of the most frequent vaginal infections, and its most common symptom is malodorous discharge. BV is associated with adverse pregnancy outcome. Treatment in early pregnancy will reduce the incidence of extreme preterm deliveries [1].

The recommended treatment regimes for BV are oral or vaginal metronidazole or vaginal clindamycin [2]. Treatment efficacy is supposedly high. In a meta-analysis, the expected cure rate after one month was 70%–80% for metronidazol [3] and 82% for clindamycin [4]. However, in clinical practice these high efficacy rates have not been observed. Based on our own review of published data, the efficacy is not more than 60% [5]. Thus it is likely that the published efficacy results are higher than the actual clinical figures. In a recent follow-up study, only 48% were BV free 12 months after therapy [6]. Consequently there is a need for a BV treatment regime that will improve the rather low efficacy of the present therapy.

Lactic-acid-producing bacilli are part of the normal bacterial microbiota of the vagina and have a physiological role in maintaining a low pH (<4.5) and protecting against invasion by other micro-organisms [7]. However, what kind of lactobacilli are commensally in the vagina is a matter of debate. Traditionally *Lactobacillus acidophilus *has been considered to be dominant. Recent studies supplementing traditional methods with genotyping through rapid amplified polymorphic DNA (RAPD) analysis, have shown the most common vaginal lactobacilli species to be *L. crispatus, L. gasseri, L. iners *and *L. jensinii *[8]. So far trials that aimed to re-implant lactobacilli as treatment for BV have not been convincing. One double-blind, randomized, placebo-controlled trial showed that women who received ovules with *L. acidophilus *were initially cured, but the next menstruation was followed by a BV relapse giving a cure rate of only 18% after 4 weeks [9]. The treatment suggestion by Reid et al. [10] to restore asymptomatic BV to lactobacilli microbiota by giving oral capsules of *L. fermentum *and *L. casei var. rhamnosus *succeeded in 37% of the treated cases compared to 13% who were given placebo. In a recent study from Nigeria, the same strains of lactobacilli ingested orally after a one-week course of oral metronidazole cured 88% of women with BV compared to 45% in the metronidazole/placebo group [11].

The use of adjuvant lactobacilli after antibiotic treatment using vaginal tampons with freeze- dried lactobacilli did not increase the efficacy. The one-month cure rate after 3 days of vaginal clindamycin followed by lactobacilli tampons cured 56% compared to 62.5% for clindamycin and placebo tampons [12].

The primary objective of this study was to investigate if supplementary lactobacilli treatment could improve the initial cure rate after vaginal clindamycin therapy, and secondly, if lactobacilli as repeated adjunct treatment during 3 menstrual cycles could lengthen the time to relapse after initial cure.

## Methods

### Study population

The study was conducted at an out-patient private gynaecological clinic in Drammen, Norway from February 2004 until May 2006. All women with symptomatic BV who visited the clinic and fulfilled the inclusion criteria were consecutively invited to participate in a prospective, double-blind, placebo-controlled study of lactobacilli supplementing the clindamycin therapy. Of the eligible patients, two rejected participation the other signed an informed consent.

Inclusion criteria were: regularly menstruating women 18 years of age or older, with normal gynaecological status, not pregnant or breast feeding, and without signs of other genital tract infections.

Exclusion criteria were: patients with hormonal IUD without regular menstruation; women with clinical Candida infection or *Trichomonas vaginalis *infection.

### Study sample

Populations of 100 patients were randomized into two groups of 50 patients. The mean age was 34.3 years with a total range of 18.8–53.6 yrs. The initial symptoms were 86% malodorous discharge, 70% increased discharge, 14% itching, and 11% burning. There were no differences in background variables. The diagnosis of BV based on Amsel criteria were 95% fulfilling the criteria: typical discharge, 94% positive whiff test, 99% had presence of clue cells (one missing data), and 100% had a pH > 4.5.

### Clinical method

At inclusion in the study, women underwent a routine gynaecological examination with non-lubricated speculum including vaginal ultrasound. A sample of vaginal secretion was analysed for vaginal pH using special pH strips (range 3.8–5.0). The BV diagnosis was based on Amsel criteria [13], i.e. fulfil at least 3 of 4 criteria: thin homogenous discharge, vaginal pH above 4.5, positive amine test, presence of clue cells during microscopical investigation. Any presence of motile curved rods, *Mobiluncus *was recorded during this first visit. The motile bacteria had to be observed in active motion in an opposed direction to the rest of the sample. Vaginal fluid was sampled and air-dried. At inclusion, vaginal samples for *Chlamydia trachomatis *infection were done with strand-displacement amplification (CT amplified DNA assay; Becton-Dickinson) according to the local laboratory routine. Samples for *Neiseria gonorrhea *were done only when there was medical reason to suspect its presence.

### Treatment

After signing the informed consent document, the participants were given a seven-day course of daily 2% vaginal clindamycin cream (Pfizer Norway Ltd) directly followed by vaginal gelatine capsules containing 10^8–9 ^freeze-dried lactobacilli or placebo capsules of identical appearance, sufficient for 10 days or until menstruation commenced; these capsules were randomized in blocks of 10. After each menstruation, the treatment with vaginal lactobacilli capsules or placebo was repeated during 10 days for three cycles. Thus the treatment regime included one treatment course with clindamycin followed by four lactobacilli/placebo courses, one within the same menstrual cycle and the other during the next three consecutively cycles.

### The trial drugs

The lactobacilli used are commercially available EcoVag^® ^Vaginal capsules (Bifodan A/S, Denmark) containing two types of lactic acid bacteria *L. gasseri *(Lba EB01-DSM 14869) minimum of 10^8–9 ^CFU/capsules and *L. rhamnosus *(Lbp PB01-DSM 14870) minimum of 10^8–9^CFU/capsules. Both are lactobacilli strains cultured from healthy women in Norway. Other ingredients: lactitol monohydrate, gelatine, cornstarch, xanthan gum, glucose anhydrous, titanium dioxide and magnesium stearate.

The placebo vaginal capsules of identical appearance contained sorbitol, gelatine, potato starch, and magnesium stearate.

### Follow up

After each menstruation, the patient self-swabbed a specimen of vaginal fluid and stroked the cotton tip on a glass slide: the sample was then air dried and submitted in a sealed envelope to the clinic according to a previously described method [12]. The vaginal sample was collected after cessation of bleeding and before starting of treatment with the vaginal capsules. Women who were considered still to be infected with BV after the first menstruation were re-treated with vaginal clindamycin and excluded from the study. The remaining participants were asked to continue to swab vaginal samples and insert vaginal capsules (lactobacilli or placebo) after each menstruation. The nurse affiliated with the study conducted a telephone interview with each participant to document treatment complications and concomitant medication. If the patient had failed to submit a sample of vaginal fluid at the right time, the sample was recorded as missing and she was reminded to send in a new sample after the next menstruation. Ideally, a total of 6 vaginal samples was collected from each participant. After four menstrual cycles, the participant was scheduled for a follow up visit to the clinic. This visit was booked to take place at least one menstruation after the final treatment with lactobacilli/placebo. Normally the vaginal samples were taken once every 28 days and the follow up time of 6 menstrual cycles thus would be 5 and half months but for some women, 6 menstrual cycles exceeded 6 months. Therefore, we report this 6-month follow up as number of BV free days between cured and relapse, as well as menstrual cycles.

### Microscopical investigation

Each vaginal sample was rehydrated with normal saline solution and surveyed under a phase contrast microscope with 400× magnification and an area of 0.016 mm^2^. A rough count of bacteria was recorded as well as the presence of clue cells together with number of vaginal leucocytes. Each slide was classified by two methods: according to Hay/Ison classification [14,15] and to Nugent [16]. Dual classifications were made for a comparative study, the findings of which will be published elsewhere.

### Definition of cure

The treatment was considered a cure when the Hay/Ison score was 1, a score of 2 was designated as improved and a score of 3 was classed as treatment failure. A Hay/Ison group of 0 and 4 were regarded as equal as a score of 1 during the first follow up periods. Treatment efficacy is based on reported both as cure after one month, and as length of time to relapse illustrated with Kaplan-Meier survival analysis, i.e. as number of BV free days between cured and relapse, and as menstrual cycles. Re-treatment with clindamycin was given in those cases with microscopical evidence of BV i.e. a Hay/Ison score of 3.

We had the possibility to grow vaginal culture to ascertain if the lactobacilli recovered from certain participants were the same strain as those provided via the lactobacilli capsules. This investigation was done by the Statens Serum Institute (SSI) in Copenhagen, Denmark without any knowledge on their part of the treatment the women had received.

### Study design

The initial part of the study was open, whereby patients were treated with vaginal clindamycin for seven days; it continued thereafter as a double-blind, randomized, placebo-controlled trial with parallel group design. The patients were equally allocated 1:1 to either lactobacilli capsules or placebo capsules of identical appearance by block randomization with a fixed block size of 10. The capsules were given for 10 days during three consecutive menstrual cycles.

### Statistical methods

Not all patients collected a vaginal fluid sample every month, but all who sampled at least once were included in the analysis. In case of missing data during the follow up, the procedure "last observation carried forward" (LOCF) was used. In case of missing data from a visit for a relapsed patient, the observation from the following visit was transferred back. This procedure was used in order not to over-estimate the effect. The variable "days from cured to relapse" has been calculated in days and is given by Kaplan-Meier plot. Analysis using the number of menstrual cycles instead of days from cured to relapse was also done. Additionally, the proportional HR ratio with 95% confidence interval (CI) is given [17] and Fisher exact test. All comparisons between and within groups were performed two-tailed with a significance level of 5%.

All study personnel were blinded to the treatment throughout the study and all the data were collected and recorded in data sheets before the code of the active or placebo treatment was added. Thereafter no changes in the data sheet were allowed.

A difference in relapse rate between the lactobacilli group and the placebo group of at least 20% was considered clinically relevant. Power analyses with a significance level of 5% and a power of 80%, resulted in at least 46 patients having to be included in each of the two treatment arms to show a 20% difference.

All the analysis in this study was performed in accordance with the principal of intent to treat (ITT).

### Ethical

The study was approved by the southeast regional Ethics Committee in Oslo, Norway. To recruit women with BV, advertisements in local newspapers requested women suffering from malodorous discharge to contact the study group. All patients did signed a written informed consent document.

## Results

The initial cure after the first menstruation was 64% (32/50) in the lactobacilli group versus 74% (37/50) in the placebo group (p = 0.12). However any patient with missing or unclassified smears at the initial visit who continued the study and whose next smear indicated a cure was included in the cured group. The cure rate would thus be 74% (37/50), (95% CI: 59.7 – 85.4) in the lactobacilli group versus 78% (39/50), (95% CI: 64.0 – 88.5) in the placebo group (p = 0.82). By omitting two of the patients in the lactobacilli group who reported that they did not take any vaginal capsules, the initial cure rate would be 37/48 (77%) in the lactobacilli group. Of the remaining 24 women, 15 were re-treated as failed treatment cases and nine dropped out of the study.

**Figure 1:**
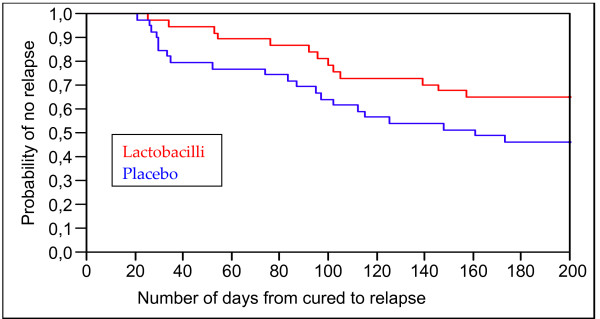
Time from cured to relapse of BV in days. The results are given as a Kaplan-Meier plot expressing the probability of no relapse before the given day. The lactobacilli-treated women have a significantly longer time until relapse with a log rank p = 0.027

**Figure 2:**
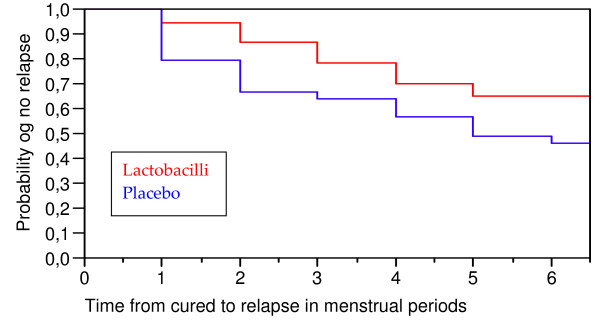
Time from cured to relapse of BV in menstrual months. The results are given as a Kaplan-Meier plot expressing the probability of no relapse before the given month. The lactobacilli-treated women have a borderline-significant longer time until relapse with a log rank p = 0.06.

The 76 women initially classified as cured were now followed until their 6^th ^menstrual cycle or until relapse. There was a significant difference (p = 0.027) in favour of the lactobacilli treated women in time duration from cured to relapse which is illustrated in Kaplan-Meier plot (Figure 1). Expressed as number of menstrual periods from cured to relapse, the findings showed a similar curve that did not reach significance (p = 0.06) (Figure 2).

At the end of the study, 64.9% (24/37) of the lactobacilli treated women were pronounced cured compared to 46.2% (18/39) of the placebo treated women giving a significant HR ratio of 0.73 (95% CI: 0.54–0.98) (p = 0.042) (see Flowchart in Additional file 1).

The presence of motile curved rods Mobiluncus showed a tendency to affect the results since there were lower cure rates among women who exhibited mobiluncus in the wet smear at the first visit (Table 1). However this difference did not reach statistical significance (p = 0.14 Fisher exact test).

**Table 1:** The frequency of relapse related to the presence of motile curved rods, mobiluncus at the inclusion visit

Mobiluncus	Relapse	No relapse	Total
No	19 (40%)	29	48
Yes	16 (57%)	12	28

Total	35	41	76

P = 0.14 Fisher exact test.

Ten patients had taken other antibiotics during the study period, six before the first follow-up visit, four from the lactobacilli group and two from the placebo group. The antibiotics were given for Chlamydia infection (2), for upper respiratory infection (3) and for urinary tract infection (1). The exclusion of these patients did not affect the study result. Two of the patients who took antibiotics did so after their relapse. Two patients took antibiotics that could have influenced the results. One woman given placebo received broad-spectrum antibiotics because of upper respiratory infection half way through the study. Her BV was cured after the antibiotics; the other patient did not show any effect of the antibiotics.

Totally 146 patients came to our clinic with malodorous discharge. Of these, 102 fulfilled the inclusion criteria although two decided not to join the study, 10 had a BV infection but did not have regular menstrual periods, 6 did not fulfil all of the Amsel criteria, 3 had other diagnosis, 9 had normal discharge, and 18 (38%) had normal pH and normal lactobacilli seen in the smear together with *Candida albicans *blastospores and mycelia. For these women, treatment with fluconazole successfully reduced the symptomatic malodour.

A total of 14 patients in the lactobacilli group and 12 in the placebo group reported adverse events (AE) during the study. The reported events were headache, menorrhagia, haemorrhoids, influenza, bronchitis, whiplash, asthma, and urinary tract infection. The study did not detect any difference between the two groups regarding type nor frequency of AE. Among the AE reported as probable or almost certain was one woman who stopped treatment because of vaginal discomfort due to suspected allergy in lactobacilli group and another due to itching in the placebo group. Five patients in the lactobacilli group and four patients in the placebo group were treated with antimycotic drugs for symptomatic candida infection.

It was possible to culture only seven lactobacilli strains. Of these strains, four were *L. gasseri *species, all from women treated with lactobacilli. These four strains were tested using 500 bp sequences in the 16S V1 region with 100% concordance to the original strain given to the women.

## Discussion

This is the second study to show that supplement therapy with two different lactobacilli strains could improve the efficacy of the BV treatment. The cure rate improved at the 6^th ^month from 46% to 65%, a nearly 20% difference that may be of clinical importance. There was, however no improvement in the initial cure rate. The ITT cure rate was 64% (32/50) in the lactobacilli group versus 78% (39/50) in the placebo group. However any patient with missing or unclassified smears at the initial visit who continued the study and whose next smear indicated a cure was included in the cured group and by omitting two of the patients in the lactobacilli group who reported that they did not take any vaginal capsules, the initial cure rate would be 37/48 (77%) in the lactobacilli group, thus there are no difference between the lactobacilli and the placebo group in the initial cure. This cure rate (77–78%) is higher than earlier reported [5] but one reason could be that we report the cure after the first menstruation and not after 28 days. The first menstruation could occur already after 14 days. Another hypothesis is that the antibiotic effect of clindamycin lasts longer than the seven days of treatment. That is feasible if clindamycin concentrates in the vagina though a counter-current circulation similar to that which has been demonstrated for penicillin [18]. In such case, the antibiotic effects of clindamycin would still be present when the first lactobacilli capsules are introduced. The lactobacilli strains are sensitive for antibiotics such as clindamycin. This could explain that there was no difference in the initial cure rate. A broad spectrum of antibiotics such as clindamycin was chosen as the trial drug since it eradicates all native lactobacilli strains making it possible to introduce new strains of lactobacilli; this is not the case for metronidazole which has no antibiotic effect on the lactobacilli. Presumably it is also possible that the strains used in this study could be upgraded to a strain that more rapidly will increase the restoration of the normal vaginal microbiota.

In the active capsules, the excipients lactitol and glucose were chosen both as diluents and as nutritive growth medium for the lactobacilli. Since the placebo capsules should not contain nutritive growth medium, sorbitol was used together with potato starch. Potato starch was selected instead of cornstarch, in the placebo capsules due to our experience that it gives the best capsule-filling ability when combined with sorbitol. The lactobacilli and the placebo capsules had identical appearance. Although we cannot be certain, we do not believe that the active excipients in the placebo capsules would affect the results of the study.

With the aim of reducing the number of relapsing patients, Sobel et al. started to treat women with recurrent BV with vaginal metronidazole gel for ten days rather than the normal five days [19]. Women who responded to this therapy were treated with metronidazole gel or placebo twice weekly for a 4-month period, followed by another 3 months without therapy – a study design very similar to our own. At 4 months, 75% were cured in the treatment group compared to 59% in the placebo group, but after 7 months, only 49% in the treatment group versus 25% in the placebo group were still BV free. Our results with 65% cured in the lactobacilli treated group and 46% in the placebo group is slightly better. There is however a difference between the two studies. Sobel et al. deliberately recruited women with recurrent BV as opposed to our study which had a random recruitment. However our material included many women who had been treated for BV before entering the study; also it also included participants who were treated for BV for the very first time. Based upon the result of this study it can be concluded that the suppressive treatment with lactobacilli is not better than suppressive treatment with metronidazole gel, but falls within the same range. However, there are ecological advantages to using lactobacilli instead of antibiotics for longer duration. Maybe a combination of both treatment regimes should be used in the most therapy-resistant cases. Results from the Nigerian study where the use of oral lactobacilli improved the cure by 40% after 30 days are very encouraging [11].

One must bear in mind that we have not inquired about any new sexual partner or partners nor have we treated any of the sexual partners. However, in our study group there are very few other sexually transmitted diseases as we had no case of *T. vaginalis *infection or *N. gonorrhoea*, and only 3 patients with *C. trachomatis *infection. Whether or not BV should be regarded as a sexually transmitted disease is controversial and a matter of major professional debate as was discussed in a previous review [20]. Still the question arises from patients with recurrent BV whether the sexual partner should be treated simultaneously or not. There is no documentation for this treatment approach in the literature [5] although many clinicians will treat the partner of the infected patient.

We had a dropout rate of 10% in this 6-month follow up study which is statistically considered to be an acceptable figure. Sobel et al. had 12% [19] dropouts and Bradshaw et al. had 6% [6].

The strength of our study is that all patients were recruited at the same clinic and that 98 of 100 patients were recruited by the same investigator and that all smears and all follow up were done by one investigator. Drawbacks are that we recruited women with recurrent BV as well as women with initial BV infections and we had no knowledge of the number of sex partners.

The reported adverse events are mild and transient and no difference between placebo or lactobacilli groups is indicated. Nine patients had been treated for symptomatic candida infections. As most other treatment studies for BV report that 10% of the patients have a symptomatic candida infection, our results are in concordance with other studies [5].

The culturing of vaginal flora sampled from cured patients was disappointing. Of seven lactobacilli strains cultured, four were *L. gasseri*, the same species given to the women in the treatment. However, as we had only sequenced 500 bp in the V1 region of the 16S, we can not with certainty claim that this lactobacilli strains is the exactly the same as the *L. gasseri *(Lba EB01-DSM 14869) given to the women. One possible reason that we only could grow seven lactobacilli cultures could be that the median transportation time from the clinic to the microbiological laboratory in Copenhagen was seven days. This is too long. The shipping was done by regular mail. Further studies have to be done to assure that lactobacilli introduced in treatment can colonize the vagina.

Our results indicate that it takes a long time for some women to suffer a relapse. Some patients had an increase in number of bacteria per vision field with Gardnerella morphotype from 0 at first month to 50, 100, 500, 2000 and finally 4000 at 6 months when the relapse was obvious. This could indicate that this is not a new infection but rather an infection that was suppressed to a level below the detection threshold of our clinical methods. With slow growing bacteria such as Mobiluncus this could quite feasible. Our study is the first to report a tendency to lower cure rate among women harbouring Mobiluncus as opposed to women without any motile curved rods.

We used Hay/Ison scoring for the diagnosis of BV [14,15] as we earlier encountered limitations in the use of Nugent scoring. This particularly applies to treatment studies and is even more pronounced when clindamycin vaginal cream is the selected treatment. Clindamycin vaginal cream eradicates almost all bacteria in the vagina, and greatly reduces the lactobacilli morphotype bacteria, giving the Nugent score of around 4. This means that the smear can be incorrectly interpreted as demonstrating intermediate flora. On the other hand, if there are only five Gardnerella morphotype bacteria per vision field, the Nugent score would increase from 0 to 3 even when there are more than 500 lactobacilli morphotype bacteria [21]. The Hay/Ison [14,15] classification is better adapted to follow up treatment results. This must be taken into consideration when discussing treatment efficacy.

Even though this was not in the protocol for our study, symptomatic malodour has not to our knowledge been reported as symptoms of candida infections before.

## Conclusion

In conclusion, this study shows that supplement treatment with two different strains of probiotic lactobacilli does not improve the efficacy of BV therapy during the initial first month. After lactobacilli treatment given during three menstrual cycles, there is a significant improvement in "time to relapse" so more women remain cured, i.e. are BV-free, after 6 months.

## Abbreviations

Adverse events (AE)

Bacterial vaginosis (BV)

Confidence interval (CI)

Hazard risk ratio (HR)

Intrauterine device (IUD)

Intent to Treat (ITT)

Last observation carried forward (LOCF)

Rapid amplified polymorphic DNA (RAPD)

Statens Serum Institute in Copenhagen, Denmark (SSI)

## Competing interests

P-G Larsson has received payment for lectures and funding for trials from Pharmacia Sweden Ltd, now merged into Pfizer Sweden Ltd. Stig Larsen has received payment for statistical analysis from Bifodan AS Denmark and Kjeld R Ryttig has received payment for study monitoring from Bifodan AS Denmark. Babill Stray-Pedersen has received payment for an earlier study about lactobacilli from Bifodan AS Denmark.

## Authors' contributions

P-GL, BS-P and KRR contributed to design. P-GL, collection of data, P-GL analysis of air-dried smears and P-GL, BS-P, KRR and SL contributed to interpretation of results, statistical analysis and writing of the study report. All authors have read and approved the final manuscript.

## Pre-publication history

The pre-publication history for this paper can be accessed here:

http://www.biomedcentral.com/1472-6874/8/3/prepub

## Supplementary Material

Additional File 1 Flowchart. Click here for file
