# Supplementary material for: Human lactobacilli as supplementation of clindamycin to patients with bacterial vaginosis reduce the recurrence rate; a 6-month, double-blind, randomized, placebo-controlled study
Source: BMC Womens Health. 2008 Jan 15;8:3. doi: 10.1186/1472-6874-8-3 (PMC3225869; doi:10.1186/1472-6874-8-3)
Supplement: Additional File 1 — Flowchart. [file 1472-6874-8-3-S1.doc]

**The Consort Flowchart**

Assessed for eligibility

(n= 148)

Excluded

(n=48)

Not meeting inclusion criteria

(n=46)

(n=10) BV but no menstruation

(n=6) BV but only 2 Amsel

(n=18) Candida

(n=8) Normal discharge

(n=3) Other diagnosis

Refused to participate

(n=2)

**Enrollment**

**Allocation**

**Analysis**

**Follow-Up**

Analyzed

(n= 37)

Cured after 6 month

(n=24)

Lost to follow-up

(n= 3)

Not cured, new treatment

(n= 10)

#

Allocated to lactobacilli treatment

(n= 50)

Received allocated intervention

(n=50)

Did not receive allocated intervention

(n= 0)

Lost to follow-up

(n= 6)

Not cured, new treatment

(n= 5)

Allocated to placebo treatment

(n= 50)

Received allocated intervention

(n= 50)

Did not receive allocated intervention

(n= 0)

Analyzed

(n=39)

Cured after 6 month

(n=18)

Randomized and treated with clindamycin vaginal cream for 7 days
